# In Vitro Biofouling Performance of Boron-Doped Diamond Microelectrodes for Serotonin Detection Using Fast-Scan Cyclic Voltammetry †

**DOI:** 10.3390/bios13060576

**Published:** 2023-05-25

**Authors:** Bhavna Gupta, Mason L. Perillo, James R. Siegenthaler, Isabelle E. Christensen, Matthew P. Welch, Robert Rechenberg, G M Hasan Ul Banna, Davit Galstyan, Michael F. Becker, Wen Li, Erin K. Purcell

**Affiliations:** 1 Neuroscience Program, Michigan State University, East Lansing, MI 48824, USA; guptabh2@msu.edu; 2 Department of Biomedical Engineering and Institute for Quantitative Health Science and Engineering, East Lansing, MI 48824, USA; perillom@msu.edu (M.L.P.); chris897@msu.edu (I.E.C.); welchma7@msu.edu (M.P.W.); wenli@msu.edu (W.L.); 3 Fraunhofer USA Center Midwest, Coatings and Diamond Technologies Division, East Lansing, MI 48824, USA; jsiegenthaler@fraunhofer.org (J.R.S.); rrechenberg@fraunhofer.org (R.R.); dgalstyan@fraunhofer.org (D.G.); mbecker@fraunhofer.org (M.F.B.); 4 Department of Electrical and Computer Engineering, Michigan State University, East Lansing, MI 48824, USA; bannag@msu.edu

**Keywords:** biofouling, boron-doped diamond, fast-scan cyclic voltammetry, serotonin, carbon fiber, microelectrodes, neurotransmitter detection

## Abstract

Neurotransmitter release is important to study in order to better understand neurological diseases and treatment approaches. Serotonin is a neurotransmitter known to play key roles in the etiology of neuropsychiatric disorders. Fast-scan cyclic voltammetry (FSCV) has enabled the detection of neurochemicals, including serotonin, on a sub-second timescale via the well-established carbon fiber microelectrode (CFME). However, poor chronic stability and biofouling, i.e., the adsorption of interferent proteins to the electrode surface upon implantation, pose challenges in the natural physiological environment. We have recently developed a uniquely designed, freestanding, all-diamond boron-doped diamond microelectrode (BDDME) for electrochemical measurements. Key potential advantages of the device include customizable electrode site layouts, a wider working potential window, improved stability, and resistance to biofouling. Here, we present a first report on the electrochemical behavior of the BDDME in comparison with CFME by investigating in vitro serotonin (5-HT) responses with varying FSCV waveform parameters and biofouling conditions. While the CFME delivered lower limits of detection, we also found that BDDMEs showed more sustained 5-HT responses to increasing or changing FSCV waveform-switching potential and frequency, as well as to higher analyte concentrations. Biofouling-induced current reductions were significantly less pronounced at the BDDME when using a “Jackson” waveform compared to CFMEs. These findings are important steps towards the development and optimization of the BDDME as a chronically implanted biosensor for in vivo neurotransmitter detection.

## 1. Introduction

Neurotransmission, or the cellular communication among neurons, is driven by both chemical and electrical impulses [1]. Chemical interactions take place when a cell releases neurotransmitters that are detectable by surrounding cells and/or itself [2]. A commonly used method to study neurotransmitter release is fast-scan cyclic voltammetry (FSCV), which allows for the detection of electrochemically active compounds on a sub-second timescale. FSCV is a background-subtracted technique that typically employs carbon fiber microelectrodes (CFMEs) to repeatedly apply brief voltage waveforms to induce oxidation and reduction in analytes of interest. The generated current from the movement of electrons at specific applied potentials allows for identification of the neurotransmitter and the resultant concentration based on the measured current magnitude. Over the last thirty years, FSCV has been developed and optimized for the detection of several common neurotransmitters, including serotonin (5-HT), dopamine (DA), norepinephrine (NE), histamine, and adenosine [3,4,5,6,7,8,9,10,11].

Recently, our team developed an all-diamond, boron-doped diamond microelectrode (BDDME) for electrochemical measurements [12,13]. Unlike other BDDMEs which are grown on tungsten or platinum metals and insulated with a polymer and CFMEs, this all-diamond electrode is freestanding, insulated with polycrystalline diamond (PCD), and batch-fabricated using wafer processing techniques. Wafer fabrication allows for ease and flexibility to produce numerous custom-fabricated electrode shapes and geometries on a single wafer, all having extremely similar performances, and removes some of the human error during traditional hand fabrication techniques such as those for CFMEs and other BDDMEs [14,15,16,17]. Several studies [12,18,19,20,21,22] have shown BDD to be an extremely versatile material for electrochemical applications due to its (1) wide working potential windows; (2) lower background currents; (3) good mechanical and chemical stability; (4) good electrochemical activity without pre-treatment; and (5) resistance to fouling. However, the nature of the BDD surface in comparison to CFMEs can also yield reduced sensitivity and slower electron transfer kinetics [23,24]. Nonetheless, numerous opportunities remain to more effectively leverage the potential benefits of BDDMEs, and optimization of the applied waveform is an easily implemented first approach to improve results.

Several waveform parameters, including the scan rate, holding potential, switching potential, and frequency, are known to influence the detected current at CFMEs [25]. Much previous work has driven the development of CFMEs to sensitively and selectively detect neurotransmitters of interest; for example, the standard waveform swept from −0.4 V to 1.3 V back to −0.4 V at 400 V s^−1^ and applied at 10 Hz is widely utilized today to detect DA [25]. Similarly, Jackson et al. (1995) developed the N-shaped waveform starting at 0.2 V to 1.0 V to −0.1 V at 1000 V s^−1^ and applied at 10 Hz, specifically to increase and isolate the 5-HT oxidation peak current, while minimizing electrode fouling [26]. Waveform optimization tailored the electrochemical response of 5-HT on the CFMEs surface and has shown that: (1) the rate of adsorption of 5-HT is higher with an N-shaped waveform compared to the triangular waveform; (2) the 5-HT current amplitude is 10 times greater when scanning at 1000 V s^−1^ compared to 100 V s^−1^; (3) an interferent electrochemical couple is less apparent at the faster scan rate of 1000 V s^−1^, and; (4) holding the potential at 0.2 V minimized interference by 5-HT oxidation byproducts which can build-up and polymerize on the electrode surface [26,27].

Modified FSCV waveform parameters provide insight into analyte detection and electrode surface interactions. With the triangular waveform at CFMEs, Heien et al. (2003) demonstrated increased sensitivity of DA and other neurotransmitters, including 5-HT, by extending the switching potential from 1.0 V to 1.4 V [28]. Recently, Venton’s group investigated an extended version of the Jackson waveform at CFMEs to attain low electrode fouling and higher sensitivity for measurements in vivo [29]. In particular, the switching potential of the Jackson waveform was extended to 1.3 V, so that the CFME surface could be constantly regenerated [30]. The Jackson waveform was determined to be the most selective for 5-HT, while the extended waveform had increased electrode sensitivity [29]. A key advantage of higher switching potentials is CFME surface activation, facilitated by the breakage of carbon–carbon bonds and addition of edge plane sites to promote analyte adsorption and surface cleaning at the electrode [25,30,31]. In an in vivo setting, such extended waveforms or higher switching potentials could be advantageous where electrode fouling and/or selectivity are prominent issues [29].

A major recognized challenge of in vivo neurotransmitter detection at the CFME is biofouling: the adsorption of biomolecules or proteins at the inserted electrode [25]. Implantation of a chronic electrode facilitates a cascade of immune response in the tissue [32,33,34]. Protein deposits on the electrode surface can disrupt analyte adsorption, slow electron transfer, and interfere with voltammetric performance [25,34,35,36]. The detection of 5-HT in vivo is especially challenging because of the added burden of oxidizable, reaction-specific side-products that irreparably foul the CFME surface [27]. The Swain group has reported significantly reduced 5-HT fouling on BDDMEs with amperometric detection compared to bare CFMEs [37] and Nafion-coated CFMEs [38]. The sp^3^ carbon structure, extended π-electron system, and fewer carbon–oxygen surface groups make the BDD resistant to high adsorption of molecules, potentially resulting in reduced fouling at the electrode surface [37,38].

In this work, we sought to characterize the in vitro FSCV electrode behavior and biofouling performance of our freestanding BDDME compared to the traditional CFME. First, we report on 5-HT responses at the BDDMEs and CFMEs over a range of FSCV parameters such as scan rate, holding potential, switching potential, frequency, and concentrations; this work is an extension of our previous conference proceeding [39]. Second, we studied the biofouling effects on the 5-HT current at both electrodes with the standard waveform. Third, we investigated biofouling-induced changes to 5-HT responses at both electrodes with the Jackson waveform. We found that the BDDME showed lower electrode fouling with increasing or changing switching potentials, frequency, and analyte concentrations, in comparison with CFMEs. Biofouling effects were significantly less pronounced at the BDDME with the Jackson waveform compared to CFMEs. The CFMEs maintain higher sensitivity and excellent LODs for 5-HT in comparison with BDDMEs for all conditions. These experiments are important steps towards optimizing the detection performance of the BDDME for in vivo neurotransmitter sensing applications.

## 2. Materials and Methods

### 2.1. Chemicals

All chemicals were purchased from Sigma-Aldrich, Inc. (St. Louis, MO, USA) and Fisher Scientific International, Inc. (Hampton, NH, USA). Stock solutions of 1 mM 5-HT were prepared in 1 mM perchloric acid and used within 24 h to prevent solution degradation. Diluted solutions of 5-HT were prepared in artificial cerebrospinal fluid (aCSF) (pH 7.4; 20.68 mM Trizma hydrochloride, 4.32 mM Trizma Base, 126 mM NaCl, 2.5 mM KCl, 1.2 mM NaH_2_PO_4_, 2.4 mM CaCl_2_, 1.2 mM MgCl_2_). Solutions of 1 mM ferrocene carboxylic acid (FcCOOH), a highly electroactive compound with a well-documented redox response with FSCV, were prepared in aCSF and used to test for optimal placement of microelectrodes in the flow injection setup before measurements were recorded. For all biofouling experiments, a 4% solution of bovine serum albumin (BSA; 40 gL^−1^ in aCSF, pH 7.4) was freshly prepared before electrode soaking. All solutions were prepared with ultrapure water: 18.2 MΩ.cm, TOC < 5 ppb (Barnstead™ GenPure™ xCAD Plus Ultrapure Water Purification System, Thermo Scientific, Waltham, MA, USA).

### 2.2. Carbon Fiber Microelectrode (CFME) Fabrication

CFMEs were constructed similarly to previously reported methods [30]. Briefly, 7.4 µm Ø, unsized, AS4 carbon fibers (Hexel, Stamford, CT, USA) were aspirated into glass capillaries (World Precision Instruments, Sarasota, FL, USA) using a vacuum pump. These capillaries were pulled with a vertical micropipette puller (Stoelting Co., Wooddale, IL, USA). An electrical connection was made by coating 32 AWG wire wrapping wire with PELCO conductive carbon-based glue (Ted Pella, Inc., Redding, CA, USA) and inserting it into the open end of the capillary and epoxying it in place. The carbon fibers were then cut to an approximate 100–150 µm exposed length measured from the glass seal. All CFMEs, unless otherwise noted, were allowed to stabilize for 20–30 min using the standard cyclic waveform of −0.4 V to 1.3 V at 400 V s^−1^, 60 Hz frequency in aCSF, and then allowed to finish stabilizing for 10 min at 10 Hz before being used for experimentation.

### 2.3. Boron-Doped Diamond Microelectrode (BDDME) Fabrication

**Figure 1:**
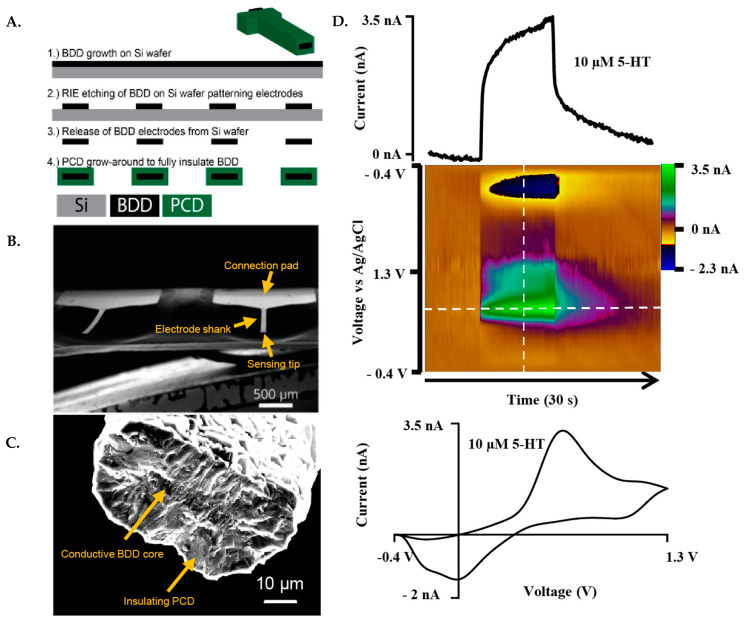
Representative fabrication scheme and FSCV response of the BDDME. (**A**). Fabrication scheme of the BDDME using wafer processing technology, in which the BDD is grown, and insulating PCD is then utilized to encapsulate the BDD core. (**B**). Scanning Electron Microscope (SEM) image of the individual, free-standing BDDME showcasing a connection pad and electrode shank. (**C**). SEM image of a BDDME sensing tip, with a BDD core area of 123 µm^2^ and polycrystalline diamond (PCD) encapsulation shell with a 15 µm thick layer. (**D**). Representative FSCV response of 10 µM 5-HT in aCSF at the BDDME with a flow rate of 750 µL min^−1^, and an applied waveform of −0.4 V to 1.3 V to −0.4 V at 400 V s^−1^ and 10 Hz repetition rate. Extracted current vs. time trace of the peak oxidation current (**top**) and cyclic voltammogram (**bottom**) showcase the electrochemical response of the BDDME when measuring 5-HT with the BDDME.

This fabrication scheme was based on a previous report [13] with some modifications, described as follows. The fabrication of the BDDMEs is a multi-step chemical vapor deposition process, which includes photolithography, metal masking, and dry etching. The fabrication scheme (Figure 1A) represents the basic key wafer processing steps. Briefly, BDD films were grown on a 4″ Ø-500 µm thick single-side polished silicon wafer using a 915 MHz microwave chemical vapor deposition reactor. Synthesis conditions include a microwave power of 9 kW, a 900 °C stage temperature, a chamber pressure of 60 Torr and a gas chemistry of 2% methane. Diborane was added to the diamond grown at a B/C ratio of 37,500 ppm to ensure conductivity. Following BDD growth, copper was thermally evaporated (Auto 306; Edward, Inc., West Sussex, UK) and patterned via photolithography (ABM-USA, Inc., Jan Jose, CA, USA), followed by wet chemical etching and reactive ion etching. The diamond electrodes were then released from the silicon using an HNA etchant with an HF:HNO_3_:CH_3_COOH composition of 5:11:6, and fully insulated with polycrystalline microcrystalline diamond using hot filament chemical vapor deposition (HF-CVD). Microcrystalline diamond was grown using a base pressure of 35 Torr and 2% methane on the freestanding released BDDMEs (Figure 1B). After deposition, the ends of the electrodes were physically cleaved to expose the BDD core, and the electrical connection was made using the conductive carbon glue (Ted Pella, Inc., Redding, CA, USA). Electroactive areas for the diamond cores ranged from 100 to 200 µm^2^ based on a 50 µm wide pattern, and a BDD growth thickness of ~2–4 µm (Figure 1C).

### 2.4. Fast-Scan Cyclic Voltammetry (FSCV) Instrumentation

A two-electrode setup (a working electrode versus a quasi Ag/AgCl reference electrode) was utilized in a custom flow injection cell for FSCV experiments. A self-constructed potentiostat with a variable gain headstage (50 nA/V, 100 nA/V, 200 nA/V, 500 nA/V, 1 µA/V) was connected to the electrode to carry out measurements. Data were collected using a NI-6363 data acquisition card and HDCV software (Version 4, Department of Chemistry, University of North Carolina, Chapel Hill, NC, USA) [40]. For all experiments, the flow injection system used a TTL voltage-controlled source to switch a six-way HPLC valve to introduce a bolus of test analyte. A flow rate of 0.75 mL min^−1^ was used to deliver aCSF buffer by a NE-1000 syringe pump (New Era Pump Systems, Inc., Farmingdale, NY, USA).

### 2.5. Waveform Parameter Investigation

The waveform factors section of the study utilized the “standard” triangular FSCV waveform, −0.4 V to 1.3 V and back at 400 V s^−1^ at 10 Hz as the baseline. The peak oxidative 5-HT current value was used to determine the effects of different waveform parameters. The following parameters were adjusted individually in the HDCV software: frequency, holding potential, switching potential, scan rate, and analyte concentration (0.025 µM up to 100 µM). For non-calibration experiments, baseline 5-HT concentrations were used for the two electrode types, 1 µM for CFMEs and 10 µM for BDDMEs due to differences in electrode sensitivity. Each data value was obtained by averaging the current response across three injections of 5-HT into the flow cell system, unless otherwise stated. An example redox response to 10 µM 5-HT at the BDDME using FSCV is presented in Figure 1D.

### 2.6. Biofouling Protocol

The in vitro biofouling of the microelectrodes was performed utilizing protocols published by Singh et al. (2011) [41], with minor changes. Briefly, electrodes were pre-calibrated with 5-HT (0.025 µM to 1 µM 5-HT for the CFMEs, and 0.2 µM to 10 µM 5-HT for the BDDMEs) and placed in BSA for ~12 h. For the duration of BSA exposure, the electrodes were fixed in a beaker containing 4% BSA in aCSF and only the tips were submerged in the solution. After removal, electrodes were post-calibrated with 5-HT, similarly within 24 h. Prior to post-calibration and following placement in the flow injection system, electrodes were positioned in the flow path and the previously measured highest concentration of 5-HT was detected to eliminate the influence of surface refreshing and pre-mature removal of any absorbed BSA (i.e., 1 µM 5-HT at CFMEs and 10 µM 5-HT at BDDMEs). Two waveforms were investigated at both CFMEs and BDDMEs to assess biofouling effects (SI Figure S1)—(1) the standard waveform and (2) the Jackson waveform. For each waveform condition, batches of freshly fabricated CFMEs and BDDMEs were employed.

### 2.7. Data Analysis

Raw data were extracted using the HDCV analysis software, and exported to a text file. Responses were then analyzed using in-house-developed FSCV analysis software for filtering and analysis. All data were filtered using a Butterworth 4th order lowpass filter at 1660 Hz for scan rates of 400 V s^−1^ and 8000 Hz for scan rates of 1000 V s^−1^. The data was also zero-phase filtered to preserve the phase shift in the current response with respect to the applied potential induced by digital filtering. Graphs were drawn and statistical analysis was carried out using Graphpad Prism.

## 3. Results

The peak oxidative 5-HT current was measured at discrete values of scan rate, holding potential, switching potential, frequency, and analyte concentration for both BDDMEs and CFMEs to compare response trends. Biofouling effects were measured with the standard DA waveform and the Jackson waveform on both electrodes.

### 3.1. Waveform Factors

#### 3.1.1. Scan Rate

**Figure 2:**
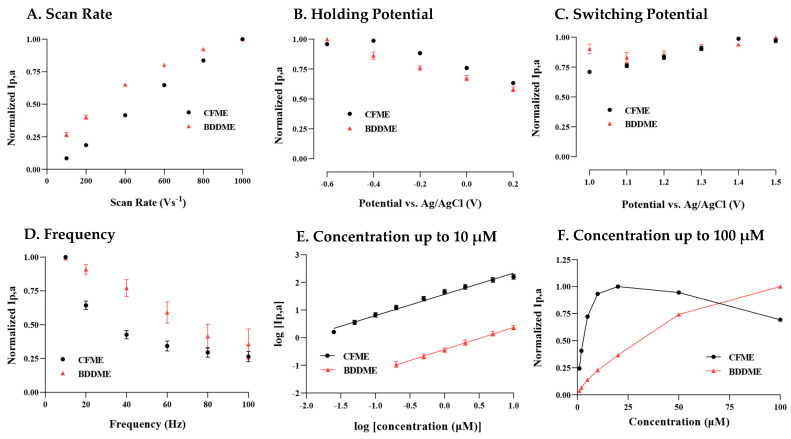
Factors of the FSCV waveform to determine the peak oxidative response for 5-HT on a CFME and BDDME. (**A**) The response of scan rate to peak oxidation, where the scan rate was modified between 100 and 1000 V s^−1^. (**B**) The peak current response from changes in the holding potential, varying from −0.6 V to 0.2 V. (**C**) 5-HT peak current response to the upper switching potential varying from 1.0 to 1.5 V. (**D**) Investigation of the applied waveform application frequency ranging from 10 Hz to 100 Hz. (**E**) The response of both the CFME and BDDME to 5-HT varies from 0.05 µM to 1 µM. The non-normalized peak current was plotted as a logarithmic response to better demonstrate the BDDME and CFME response on a comparable scale. (**F**) Concentration response from 1 µm to 100 µm, on both the BDDME and CFME to determine the upper detection ranges before sensor saturation. Note that on all plots, peak currents are normalized to the largest current, except in Figure 2E where the logarithmic of raw current and concentration are plotted. Data are represented as mean ± SEM CFMEs (*n* = 5–6) and BDDMEs (*n* = 5).

In Figure 2A, the anodic current increases linearly over the entire range of scan rates 100 V s^−1^ to 1000 V s^−1^ at the CFME, indicating an adsorption-controlled process. The BDDME’s current response is linear up to 400 V s^−1^, before beginning to plateau. When plotted as the square-root of the scan rate, the slope becomes linear, potentially indicating a diffusion-controlled process (SI Figure S2A).

#### 3.1.2. Holding Potential

Figure 2B demonstrates that the largest oxidative current was measured at −0.6 V, and the peak currents decrease with increasing positive potential for both the BDDME and the CFME. There is one exception, where the CFME has a slight increase in measured response when stepping from −0.6 V to −0.4 V before a continuing decrease.

#### 3.1.3. Switching Potential

Both the BDDME and CFME response to 5-HT differ in response to increasing switching potential (SP) (Figure 2C). The CFME has an increasing peak current response as the SP is increased up to 1.4 V, with a slight decline at 1.5 V. This trend is only observed in new CFMEs that were previously inactivated, i.e., electrodes have not been subjected to potentials larger than 1.0 V. Conversely, CFMEs that had previously experienced higher SPs show a very slight decreasing trend in response to increasing SP (SI Figure S2B). The BDDME maintains a stable anodic peak response for all SPs (with minor fluctuation, but no trend in either direction) regardless of activation and prior use.

#### 3.1.4. Frequency

Figure 2D demonstrates that both microelectrode types exhibit a decreasing peak anodic response with increasing waveform application frequency. The CFME shows an exponential decrease, while the BDDME maintains a linear decrease in peak anodic response. The BDDME application rate indicates a higher resilience in response measurements at higher scanning frequencies than the CFME, further supporting that 5-HT favors a diffusion-controlled process on a BDDME rather than adsorption.

#### 3.1.5. Concentration Lowest to 10 µM

Both BDDMEs and CFMEs maintain a linear response to increasing 5-HT concentrations from 0.025/0.2 µM (CFME/BDDME) up to 10 µM. In Figure 2E, the calibration curves for both electrodes are reported as the logarithmic of raw current vs. logarithmic of concentration to demonstrate the linear responses and measured signal variability. The signal variability between the CFME and BDDME is due to the possible difference in electroactive areas between the electrodes. The geometric surface area of the CFMEs is estimated to be 1138 to 1578 µm^2^, while the surface area for the BDDME is 123 to 200 µm^2^. The limit of detection (LOD) for the CFME, as determined from the current response of the noise (3 × standard deviation of the noise), was 0.06 µM with a non-logarithmic linear response between 0.02 µM and 0.5 µM, maintaining a sensitivity of 54.59 nAµM^−1^ (R^2^ = 0.9937). Similarly, from the noise response of the electrode, the LOD at BDDMEs was calculated to be 0.52 µM with a linear response 0.2 µM and 5 µM 5-HT and a sensitivity of 0.2901 nAµM^−1^ (R^2^ = 0.9951).

#### 3.1.6. Concentration 1 µM to 100 µM

Figure 2F demonstrates that, at concentrations of 5-HT 25 µM, both electrodes lose response linearity. However, the CFME saturates and decreases in the response from 50 µM and 100 µM compared to the BDDME, which maintains an increasing, nonlinear current response up to 100 µM of 5-HT.

### 3.2. Biofouling Effects

#### 3.2.1. Standard Waveform

**Figure 3:**
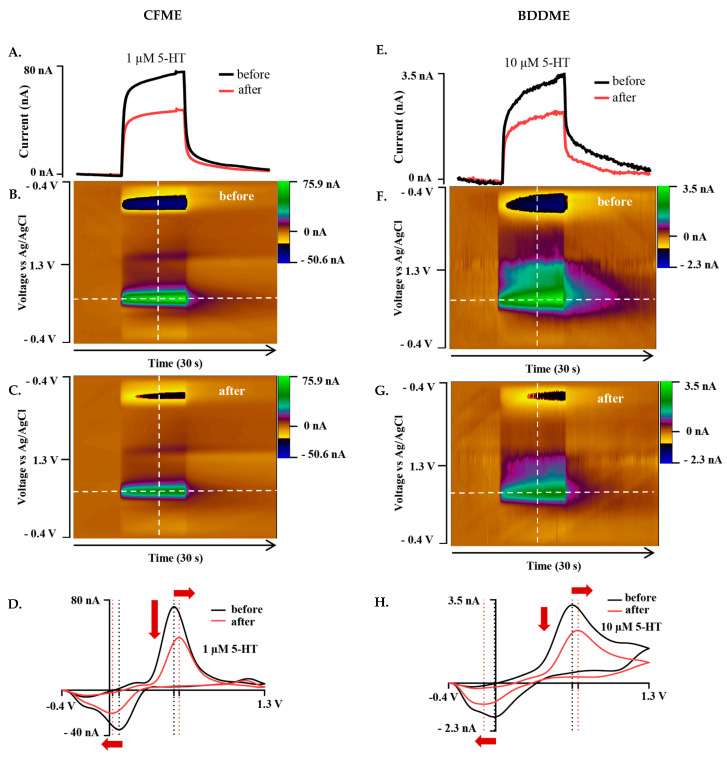
Representative biofouling effects on the 5-HT response with both the CFME and BDDME using the standard DA waveform from −0.4 V to +1.3V to −0.4 V at a scan rate of 400 V s^−1^ and 10 Hz application frequency. (**A**) Current vs. time trace extracted from the color plots (**B**,**C**) for the response of 1 µM 5-HT before and after biofouling the CFME surface. (**D**). Extracted voltammogram from the CFME response showing the change in sensitivity from fouling to 1 µM 5-HT. (**E**). Current vs. time trace extracted from the color plots (**F**,**G**). for 10 µM 5-HT measured on the BDDME from biofouling the electrode surface. (**H**). Extracted cyclic voltammogram from the color plot for the BDDME showcasing the biofouling changes to the measured response. Dashed lines on color plots indicate extracted CV and current vs. time traces. Red arrows in (**D**,**H**) indicate direction of CV peak shift after biofouling.

Biofouling-induced changes were studied at both CFMEs and BDDMEs by measuring 5-HT responses on the standard cyclic waveform (−0.4 V to 1.3 V at 400 V s^−1^ and 10 Hz) before and after exposure to BSA. From the calibration response in Figure 2E,F, the 5-HT concentrations for biofouling experiments were chosen as 1 µM at the CFME (Figure 3A–D) and 10 µM at the BDDME (Figure 3E–H). Figure 3 shows representative current vs. time traces (I vs. T), color plots, and cyclic voltammograms (CVs) for the CFME and BDDME with the standard waveform. The representative CFME in Figure 3A–D maintained a 1 µM 5-HT current response of 75.33 nA before biofouling, and a 48.07 nA (a 36.19% decrease) after biofouling after 12–14 h of soaking in BSA. Similarly, the CV after biofouling showed that the 5-HT oxidation peak decreases and shifts positively from 0.54 V to 0.59 V, while the reduction peak also decreases and shifts negatively ~0.06 V from 0.08 V to 0.025 V (Figure 3D). The representative BDDME (Figure 3E–H) measuring 10 µM 5-HT maintained a 3.52 nA oxidative peak that reduced to 2.35 nA (33% decrease) after biofouling. The 5-HT anodic peak shifted positively from 0.65 V to 0.70 V, while the reduction peak shifted negatively from −0.01 V to −0.1 V (Figure 3H).

#### 3.2.2. Jackson Waveform

**Figure 4:**
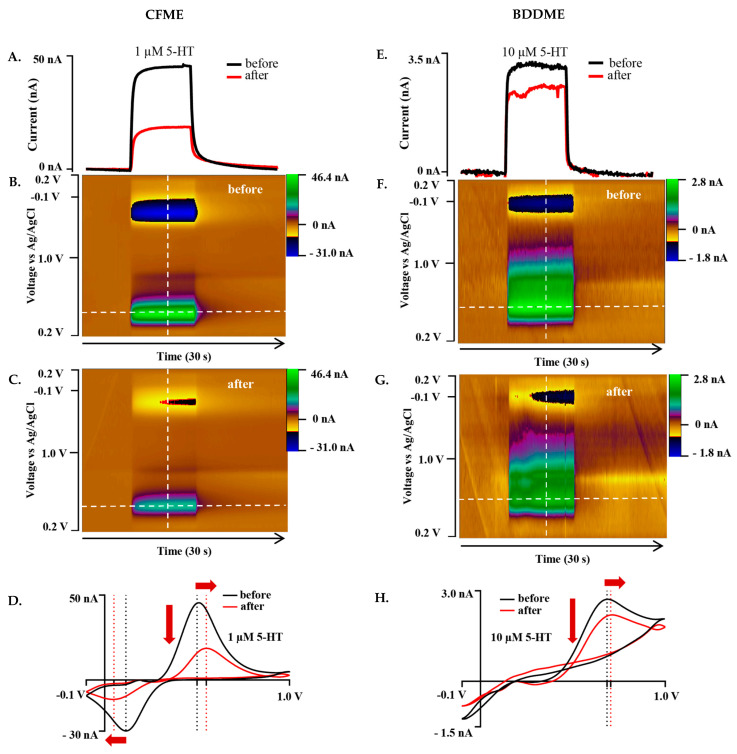
Representative biofouling effects on the 5-HT response with both the CFME and BDDME using the Jackson waveform 0.2 V to +1.0 V to −0.1 V to 0.2V at a scan rate of 1000 V s^−1^ and 10 Hz application frequency. (**A**) Current vs. time trace extracted from the color plots (**B**,**C**) for the response of 1 µM 5-HT before and after biofouling the CFME surface. (**D**). Extracted voltammogram from the CFME response showing the change in sensitivity from fouling to 1 µM 5-HT. (**E**). Current vs. time trace extracted from the color plots (**F**,**G**) for 10 µM 5-HT measured on the BDDME from before and after biofouling the electrode surface. (**H**). Extracted cyclic voltammogram from the color plot for the BDDME showcasing the biofouling changes to the measured response. Dashed lines on color plots indicate extracted CV and current vs. time traces. Red arrows in (**D**,**H**) indicate direction of CV peaks shift after biofouling.

The Jackson waveform (0.2 V to 1.0 V to −0.1 V to 0.2 V at 1000 V s^−1^ and 10 Hz), developed specifically for 5-HT measurement, was employed to understand whether waveform characteristics influence biofouling effects on both the CFME and BDDME. On both the CFME and BDDME, 1 µM and 10 µM of 5-HT were measured both before and after exposure to BSA for 12–14 h on newly fabricated electrodes (Figure 4). The CFME oxidative current response to 1 µM 5-HT decreased from 45.37 nA to 16.83 nA after BSA exposure (a 62.89% decrease) (Figure 4A–D). The CV oxidation peak shifted positively from 0.5 V to 0.55 V, and the reduction peak shifted from 0.12 V to 0.05 V (Figure 4D). The BDDME anodic response for 10 µM 5-HT decreased after exposure to BSA (Figure 4E–H) from 2.81 nA before biofouling, and reduced by 23.5% to 2.15 nA. The CV oxidative peak shifted from 0.69 V to 0.71 V (Figure 4H). Due to the increase in the applied scan rate of 1000 V s^−1^, the cathodic sweep was not resolved at the scanned potential window.

#### 3.2.3. Calibration Curves

**Figure 5:**
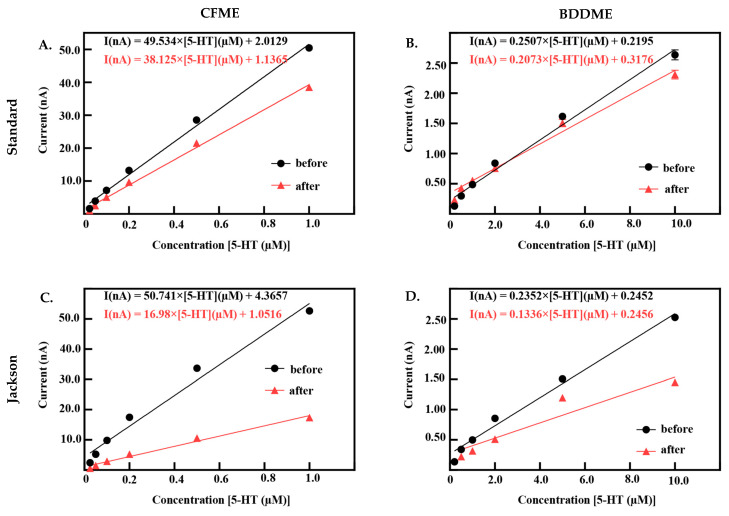
Calibration curves for both CFME and BDDMEs pre- and post-biofouling. (**A**,**B**) represent 5-HT responses measured using the standard DA waveform before and after biofouling. CFMEs (*n* = 7) and BDDMEs (*n* = 2–8). (**C**,**D**) Response before and after biofouling on the CFME (*n* = 7) and BDDME (*n* = 2–7) to 5-HT measured with the Jackson waveform. Raw currents are plotted as mean ± SEM. Note that some BDDMEs were excluded post-biofouling with both waveforms due to physical issues that resulted in a loss of electrochemical connection between the fiber and conductive pad.

Both CFMEs and BDDMEs were calibrated before and after biofouling to better understand electrode performance and recovery. Figure 5 demonstrates the linear raw current responses to increasing 5-HT concentrations pre- and post-biofouling at the CFME and BDDME with the standard and Jackson waveforms. Due to the differences in electrode surface area and response curves reported in Figure 2E,F, the 5-HT calibration concentration ranges were chosen as 0.025 µM to 1.0 µM for CFMEs, and 0.2 µM to 10.0 µM for BDDMEs. CFME responses to 5-HT concentrations before and after biofouling on the standard waveform are reported in Figure 5A. The LOD of 5-HT at the CFME before biofouling was 0.049 µM calculated from the linear best fit equation with a sensitivity of 55.58 nAµM^−1^ (R^2^ = 0.995). After biofouling, the LOD of 5-HT was maintained at 0.04 µM, but the sensitivity decreased to 42.50 nAµM^−1^ (R^2^ = 0.997). Before biofouling, the BDDMEs maintained an LOD of 0.26 µM calculated from the linear best fit equation with a sensitivity of 0.39 nAµM^−1^ (R^2^ = 0.993) (Figure 5B). After biofouling, the BDDMEs LOD for 5-HT increased to 0.83 µM with a decreased sensitivity of 0.27 nAµM^−1^ (R^2^ = 0.937) (Table 1).

When using the Jackson waveform for CFME measurements (Figure 5C), the 5-HT LOD was calculated to be 0.09 µM with a sensitivity of 64.21 nAµM^−1^ (R^2^ = 0.985). After fouling, the CFME had a decrease in sensitivity to 20.52 nAµM^−1^ (R^2^ = 0.988) with an LOD increase of 0.079 µM. The BDDME with the Jackson waveform (Figure 5D) had an LOD of 0.40 µM and a sensitivity of 0.38 nAµM^−1^ (R^2^ = 0.984). After biofouling, 5-HT LOD increased to 1.02 µM and the sensitivity decreased to 0.26 nAµM^−1^ (R^2^ = 0.907) (Table 2). It is important to note that some BDDMEs suffered physical issues during biofouling; the electrochemical connection on these electrodes was weakened, possibly due to contact pad damage or deterioration of carbon glue in the BSA soak model. These electrodes were excluded from the reported datasets.

#### 3.2.4. Statistical Analysis

**Figure 6:**
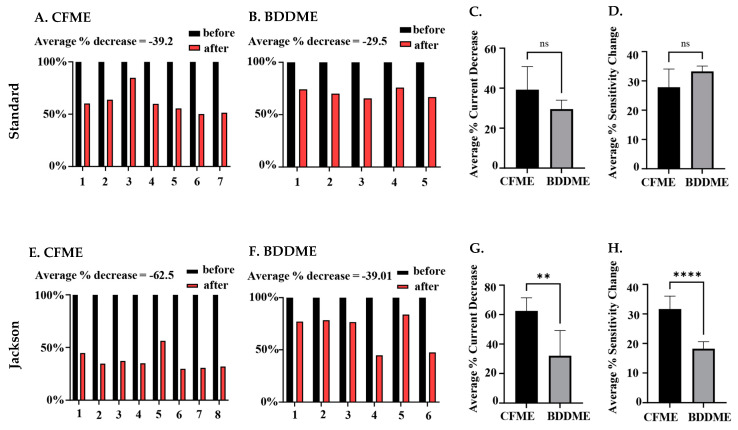
Quantification of biofouling effects on CFMEs and BDDMEs. (**A**) Individual raw current responses to 1 µM 5-HT at the CFME (*n* = 7) before and after biofouling with the standard waveform. (**B**). Individual raw current response to 10 µM 5-HT at the BDDME (*n* = 5) before and after biofouling with the standard waveform. (**C**). Comparison of average current decrease in 5-HT after biofouling between BDDMEs and CFMEs with the standard waveform; not significant, *p* = 0.0792. (**D**). Average sensitivity decrease after biofouling between BDDMEs and CFMEs with the standard waveform; not significant, *p =* 0.0885. (**E**). Individual raw current responses to 1 µM 5-HT with the CFME (*n* = 8) before and after biofouling with the Jackson waveform. (**F**). Individual raw current response to 10 µM 5-HT with the BDDME (*n* = 6) before and after biofouling with the Jackson waveform. (**G**). Average current decrease in 5-HT after biofouling between BDDMEs and CFMEs with the Jackson waveform; significant, ** *p* < 0.05. (**H**). Average sensitivity decrease after biofouling between BDDMEs and CFMEs with the Jackson waveform; significant, **** *p <* 0.0001.

The statistical comparison between both CFMEs and BDDMEs with each waveform is shown in Figure 6. On average, the current response due to biofouling (prior to post-calibration) decreased by −39.2% for the CFMEs when measuring 1 µM 5-HT (Figure 6A) and −29.5% for BDDMEs when measuring 10 µM 5-HT (Figure 6B) with the standard waveform. Although the BDDME showed less percent decrease overall, the average reduction in 5-HT response before vs. after biofouling was not significantly different between the two electrodes (Figure 6C; Welch’s *t*-test, *p* = 0.0792, two-tailed, *t* = 2.002, *df* = 8.245).

When comparing the Jackson waveform, the average current response to 1 µM 5-HT decreased by −62.5% for the CFMEs (Figure 6E) and by −39.01% for the 10 µM 5-HT on the BDDMEs (Figure 6F). The overall percentage decrease from before versus after biofouling was significantly different between the two electrodes (Figure 6G; Welch’s *t*-test, *p* = 0.0054, two-tailed, *t* = 3.964, *df* = 7.028). The sensitivity changes between CFMEs and BDDMEs were also significant (Welch’s *t*-test, *p* < 0.0001, two-tailed, *t* = 6.823, *df* = 9.737). The BDDME had less of a decrease in sensitivity than CFMEs after biofouling (Figure 6H) with the Jackson waveform. The average percentage current decrease after biofouling was greater at CFMEs with both waveforms.

## 4. Discussion

Successfully and safely maintaining chronic in vivo signals for long periods of time remains a significant challenge for implanted neurochemical sensors [42,43]. Biofouling [36], gliotic cellular encapsulation [32], insertional damage to the device and/or tissue, interferents [44], and polarization of the reference electrode [34] can all undermine the voltammetric response quality in the in vivo environment. We are developing a customizable, all-diamond, microfabricated BDDME to meet these needs, with key advantages for a chronically implanted neurochemical sensor [12]. In this paper, we characterized the current responses on both a CFME and BDDME through varied waveform parameters and biofouling conditions as necessary steps toward characterizing and optimizing detection performance for eventual in vivo usage.

The effects of varying waveform parameters (Figure 2) largely followed previously reported literature for waveform optimization on a CFME [25,44,45,46]. Elevated scan rates increased the oxidative current response linearly for the CFMEs, indicating adsorption-controlled processes at this electrode [25]. The sub-linear current response shown on BDDMEs is proportional to the square root of the scan rate, indicating that the background increases faster than the Faradaic currents and kinetics at the electrode may be diffusion-controlled [25] (SI Figure S2A). However, the reported data also support potential adsorption-controlled processes on the BDDMEs, as the peak anodic current decreased with increased application frequency of waveform application. Reduced frequency of the applied waveform allows more time for the analyte of interest to adsorb on the electrode surface, enhancing detection [25,47]. It is notable that the attenuation of current with increased frequency is less pronounced on BDDMEs than CFMEs, suggesting that the BDDME measurement of 5-HT is a dual modality measurement. As diamond, which is rich in sp^3^ carbon, lacks the adsorption sites for other carbon surfaces [48,49,50,51], further investigation is warranted on the electron transfer kinetics of the BDDME.

As the holding potential (HP) is decreased, there is a resultant increase in adsorption of 5-HT at the electrode surface [25]. This is observed in the anodic peak current, with the maximum at a HP of −0.6 V for BDDMEs. Similarly, the anodic peak current increased with reduced HP on the CFME, although the current plateaued from −0.4 to −0.6 V, which could be due to the potential window of the CFME in aqueous environments. Near −0.6 V, oxygen reduction would begin to interfere with 5-HT measurements [11,25]. As catecholamines are inherently positively charged at physiological pH, negative HPs are favorable and promote more adsorption due to the electrostatic charge difference [52]. As mentioned, 5-HT is a complex molecule that resides in a reduced state and forms side products upon oxidization that polymerize and foul the carbon fiber surface for subsequent measurements [27,29]. Previous works have shown that holding at less negative potentials mitigated this fouling effect at CFMEs [26,27]. Furthermore, very negative HPs can facilitate oxygen reduction at CFMEs that may interfere with the recorded analyte current [25]. While CFMEs require adjustments in this parameter for optimal 5-HT detection, BDDMEs may allow for more flexibility due to its resistance to fouling [37] and wide working potential [23].

When a CFME becomes activated through applied potentials above 1.0 V, the current increase is a one-time occurrence, and is non-repeatable for a given CFME. The Wightman group has shown that the CFME surface etches at potentials over 1.0 V [53] and higher switching potentials (~1.3 V) irreversibly etch the electrode surface [30]. Initial exposure to a high potential activates the CFME surface and promotes adsorption at defect sites [25,30]. Our data corroborate this effect, as electrodes that had experienced 1.4 V previously showed a decreasing current with increasing switching potential values from 1.0 V to 1.5 V (SI Figure S2B). The caveat to this phenomenon is that the CFME surface will etch away at a non-trivial rate and the surface becomes too small to be effective, particularly in a chronic setting [25,30]. Conversely, at the BDDME, the current response is relatively stable for all switching potentials regardless of prior exposure to higher potential values. Due to its carbon structure and lack of oxygen groups, the BDDME surface may not etch to the same degree as the CFME, suggesting a potential for greater stability in a chronic in vivo setting [12,54].

Analyte concentration vs. oxidative current responses are important to investigate, as detected in vivo signals can fall within the linear range of the in vitro calibrated current curves, allowing the amount of neurotransmitter release to be estimated [25]. The BDDME exhibits a linear oxidative current response from 0.2 µM to 10 µM of 5-HT, while CFME is sensitive to lower concentrations and displays linearity over 0.02 µM to 1 µM of 5-HT. The 5-HT LOD for BDDMEs was 0.52 µM and 0.06 µM for CFMEs, calculated from the linear best fit equation of the respective calibration ranges. There are two possible reasons for the higher LOD and lower sensitivity at BDDMEs compared to CFMEs: (1) the estimated geometric surface area of our BDDME (123 to 200 µm^2^) is 10 times smaller than that of the CFME (1138 to 1578 µm^2^), and (2) the sp^3^ carbon structure and lack of carbon–oxygen functional groups prevent surface adsorption of 5-HT at the electrode. The latter is a well-known tradeoff for BDDMEs, and resistance to the high adsorption of analytes has been discussed as a beneficial feature for reducing surface fouling [12,18,37]. In fact, at higher concentrations of 5-HT (1 µM to 100 µM), a fouling effect is especially evident at CFMEs and not observed on the BDDMEs (Figure 2F). Unlike at the BDDME, the 5-HT responses on the CFME began to decrease rather than plateau after 20 µM, suggesting that the surface was possibly irreversibly fouled. Jackson et al. (1995), as well as Hashemi et al. (2009), showed that 5-HT oxidation, even at 400 V s^−1^, forms byproducts that polymerize and create layers at the electrode surface [26,27], limiting electron transfer and decreasing sensitivity, thereby changing the response of the analyte with time and resulting in accelerated fouling at large concentration exposures [29]. This effect is not observed in the calibration of the BDDMEs, again suggesting potential for greater signal stability and resistance to byproduct fouling. At the same time, the smaller electroactive surface area and lower 5-HT sensitivity of the BDDME could be a major challenge for in vivo detection. Further research will focus on increasing the electroactive area to enable lower concentration detection.

While the Jackson waveform was developed to “outrun” polymerization of the byproducts of 5-HT oxidation, the in vivo environment presents additional detection barriers posed by non-specific adsorption of proteins and subsequent cellular encapsulation of the electrode surface resulting in biofouling [55,56]. As expected, our BSA soak model of biofouling reduced the detected current on both CFMEs and BDDMEs, in accordance with previous reports. Singh et al. (2011) investigated 1 µM DA response at the bare CFME before and after biofouling by exposing electrodes to a common fouling agent (Bovine Serum Albumin, BSA) and brain tissue (in vivo and in vitro) [41]. The electrode sensitivity to DA decreased significantly in each biofouling condition (60–70% reduction after in vivo brain tissue cycling) [41], which is reasonably well-aligned with our overall detected in vitro 5-HT current decrease of ~40–60% at the CFMEs (Figure 6A,E). Along with reduced sensitivity, the faradaic peaks shifted in the cyclic voltammograms and calibration curves show signal instability for all after-soak conditions in our experiments. These features are indicative of electrode fouling [21], thereby supporting the view that BSA soaking effectively replicated an in vitro biofouling effect on both CFMEs and BDDMEs.

The lower average 5-HT percent current decrease detected at BDDMEs (−39.01% decrease) in comparison to that at CFMEs (−62.5% decrease) with the N-shaped Jackson waveform (Figure 6E,F) likely reflects a combination of factors: (1) it has been suggested that BDD is less prone to biofouling [18,37,38], which may be partially attributable to its less adsorptive surface character [27], and (2) 5-HT anodic current responses are less sensitive to switching potentials from 1.0 V to 1.5 V at the BDDME compared to those at the CFME (as observed in Figure 2C and SI Figure S2B). The latter could indicate that the BDDME does not require as much surface refreshing as the CFME for accurate signal measurements in a fouled setting. Another observation to support this idea is that the BDDMEs biofouled more with the Jackson waveform (−39.01% decrease) than with the standard waveform (−29.5% decrease), but not to the same extent as the CFMEs. The CFMEs biofouled much more with the Jackson waveform (−62.5% decrease) than with the standard waveform (−39.2%). Since the Jackson waveform only scans up to 1.0 V, the CFME surface was not regenerated [30], and possibly experienced more fouling than with the standard waveform which scans up to 1.3 V. The Venton group developed an extended waveform to address this issue at CFMEs and showed reduced electrode fouling when the switching potential of the Jackson waveform was set to 1.3 V compared to 1.0 V [29].

Scanning at higher potentials may also influence electrode recovery after biofouling, as observed in our data where calibration curves for after-soak conditions were steeper with the standard waveform in comparison with the Jackson waveform for both electrodes. The curves demonstrate that after biofouling, the sensitivity to 5-HT concentrations was reduced at both CFMEs and BDDMEs. However, with continuous sweeping of waveforms, both electrodes were able to closely recover to the original current responses with the standard waveform, but neither recovered with the Jackson waveform (Figure 5). The average percent sensitivity decrease after biofouling at BDDMEs was significantly lower than that at CFMEs with the Jackson waveform (Figure 6H), further indicating that BDDMEs may not require as much surface cleaning as CFMEs post-fouling. Indeed, as summarized in Table 1 and Table 2, BDDME sensitivity decreased from 0.385 nAµM^−1^ to 0.271 nAµM^−1^ (29% decrease) with the standard waveform, and from 0.383 nAµM^−1^ to 0.260 nAµM^−1^ (32% decrease) with the Jackson waveform—relatively similar sensitivity decreases of ~30% for both waveforms The CFME sensitivity drops from 55.58 nAµM^−1^ to 42.50 nAµM^−1^ (−23% decrease) with the standard waveform, and from 64.21 nAµM^−1^ to 20.52 nAµM^−1^ (−68% decrease) with the Jackson waveform. Nevertheless, it is important to note that the CFME maintains a lower, more stable LOD for 5-HT in each condition with both waveforms.

Overall the reduced sensitivity and higher LODs of 5-HT are an important limitation of the BDDME, as evidenced in our data by the comparatively smaller peak oxidative currents relative to responses detected on CFMEs. This is an important consideration for transfer to the in vivo environment, where neurotransmitters are especially challenging to detect: in addition to fouling and interferents, levels of neurotransmitters in the intact brain are typically in the sub-micromolar range (e.g., stimulus-evoked 5-HT levels reportedly measured at 12.7 ± 1.60 nM in the rat brain [57]). Furthermore, the estimated geometric surface area of the rectangular BDDME is roughly 10 times smaller than the cylindrical CFME in this study. This could be a major challenge in vivo, as large-surface-area microelectrodes allow for sampling from numerous sites/neurons to capture a detectable signal [27]. Further, the BSA fouling conditions shifted the LOD of 5-HT to higher concentrations for BDDMEs, which could suggest that the electroactive surface was blocked due to protein adsorption at the electrode face.

Several possible modifications to the electrode could enhance results, including increasing the electroactive surface area, and modification of the surface of the diamond. The Chestek group, in their CFME-parylene fabrication, utilized a pulsed green laser to remove and burn parylene-c insulation on carbon fiber electrodes to expose even tips with increased surface areas [58]. Similarly, a laser cutting system could be employed to selectively remove PCD, creating a cylinder-style electrode of all-diamond, exposing the conductive diamond core (Figure 1E) and increasing the surface area of the electrode. Furthermore, in this study, we identified potential parameter modifications that could be used to optimize results on the BDDME in accordance with previous literature [25]. Modifications to the switching and holding potentials in waveforms, such as in the extended Jackson waveform and extended hold serotonin waveform developed by the Venton group [29], could reduce fouling and improve BDDME sensitivity to 5-HT in future studies. Similarly, experimentation with the “sawhorse” waveform devised by Kiethley et al. (2011) [59], with scan rates above 1000 V s^−1^ to increase CFME sensitivity, could help tackle low sensitivity at BDDMEs. However, a more comprehensive report on the kinetics and processes controlling analyte detection at the BDDME surface may be necessary to solidify limitations of the electrode. For in vivo 5-HT detection, it may be crucial to assess a key downstream metabolite, 5-hydroxyindoleactic acid (5-HIAA), for presence and interference, along with biofouling effects on the 5-HT oxidative current at the BDDME. Pretreatment of the BDDME with cation exchange polymers (e.g., Nafion) could further reduce fouling and isolate 5-HT responses among interferents in the in vivo environment [27].

## 5. Conclusions

This study is the first account of FSCV waveform parameter investigation and in vitro biofouling performance of the freestanding BDDME for 5-HT detection. The results from this work can guide future improvements in electrode fabrication and electrochemical detection of 5-HT at the BDDME, particularly for chronic in vivo settings. Our BDDME is unique in its design as a discrete, microfabricated device with the potential to reduce fouling and have better long-term stability in vivo. The BDDME demonstrated greater stability and reduced fouling over changing switching potentials, waveform frequencies, and analyte concentrations. Furthermore, biofouling-induced effects on the peak anodic 5-HT current were less prominent at the BDDME, especially with the Jackson waveform, compared to the CFME. Meanwhile, the CFME displayed excellent LODs for 5-HT and maintained linear responses at lower ranges of concentration in the waveform as well as biofouling experiments. The BDDME suffers from issues of low sensitivity and a small geometric surface area that could present major challenges for in vivo detection of 5-HT. The results from this work could guide modified electrode fabrication geometries and waveform strategies to optimize the performance of the BDDME as a chronic in vivo neurochemical sensor.

## Figures and Tables

**Table 1:** Summary of results for 5-HT responses before and after biofouling conditions with the standard waveform.

Electrode (Standard WF)	LOD * (µM)	Slope * (nAµM^−1^)	Measured Range * (µM)	R-Squared *	Biofouling
CFME	0.049	55.578	0.025–0.5	0.995	Before
	0.04	42.497	0.025–0.5	0.997	After
BDDME	0.26	0.385	0.2–2.0	0.993	Before
	0.83	0.271	0.2–2.0	0.937	After

* CFMEs (*n* = 7) and BDDMEs (*n* = 2–8).

**Table 2:** Summary of results for 5-HT responses before and after biofouling conditions with the Jackson waveform.

Electrode (Standard WF)	LOD * (µM)	Slope * (nAµM^−1^)	Measured Range * (µM)	R-Squared *	Biofouling
CFME	0.09	64.21	0.025–0.5	0.985	Before
	0.04	42.497	0.025–0.5	0.997	After
BDDME	0.4	0.383	0.2–2.0	0.984	Before
	1.02	0.260	0.2–2.0	0.907	After

* CFMEs (*n* = 7) and BDDMEs (*n* = 2–7).

## Data Availability

Data will be made available upon request to authors.

## Supplementary Materials

The following supporting information can be downloaded at: https://www.mdpi.com/article/10.3390/bios13060576/s1, Figure S1: Diagrammatic view of standard and Jackson waveforms; Figure S2: Further investigation of 5-HT oxidative response to scan rate at BDDMEs, and to increasing switching potential at inactivated CFMEs; Figure S3: Background + faradaic currents at CFMEs and BDDMEs; Figure S4: Response stability over 2 h at the BDDME with the Jackson waveform.
