# Supplementary material for: In Vitro Biofouling Performance of Boron-Doped Diamond Microelectrodes for Serotonin Detection Using Fast-Scan Cyclic Voltammetry
Source: Biosensors (Basel). 2023 May 25;13(6):576. doi: 10.3390/bios13060576 (PMC10296319; doi:10.3390/bios13060576)
Supplement: Supplementary file 1 [file biosensors-13-00576-s001.zip › biosensors-2374499-supplementary.pdf]

Supplementary Information

In Vitro Biofouling Performance of Boron-Doped Diamond Microelectrodes for Serotonin Detection Using Fast-Scan Cyclic Voltammetry

Bhavna Gupta [1], Mason L. Perillo [2], James R. Siegenthaler [3,4], Isabelle E. Christensen [2], Matthew P. Welch [2], Robert Rechenberg [3], G M Hasan Ul Banna [4], Davit Galstyan [3], Michael F. Becker [3], Wen Li [2,3,4] and Erin K. Purcell [1,2,4,*]

[1] Neuroscience Program, Michigan State University, East Lansing, MI 48824, USA; guptabh2@msu.edu
[2] Department of Biomedical Engineering and Institute for Quantitative Health Science and Engineering, East Lansing, MI 48824, USA; perillom@msu.edu (M.L.P.); chris897@msu.edu (I.E.C.); welchma7@msu.edu (M.P.W.); wenli@msu.edu (W.L.)
[3] Fraunhofer USA Center Midwest, Coatings and Diamond Technologies Division, East Lansing, MI 48824, USA; jsiegenthaler@fraunhofer.org (J.R.S.); rrechenberg@fraunhofer.org (R.R.); dgalstyan@fraunhofer.org (D.G.); mbecker@fraunhofer.org (M.F.B.)
[4] Department of Electrical and Computer Engineering, Michigan State University, East Lansing MI 48824, USA; bannag@msu.edu
* Correspondence: epurcell@msu.edu; Tel.: +1-517-355-3867

A. Standard Waveform (triangular)

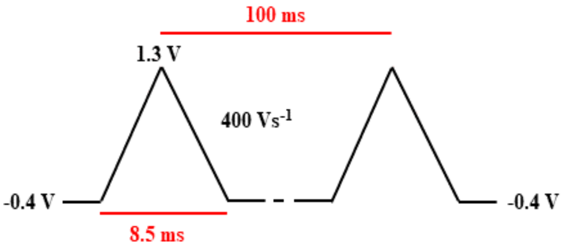

B. Jackson Waveform (N-shaped)

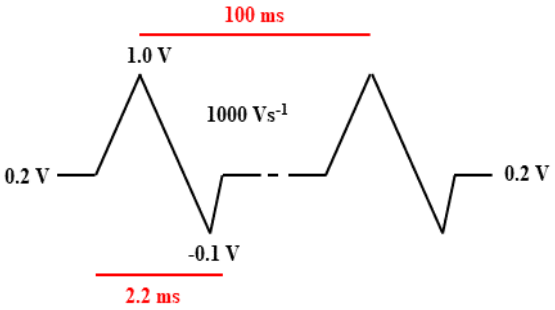

Figure S1. Diagrammatic view of standard and Jackson waveform. **A.** Standard waveform scans at 400 Vs^{-1} while holding at a negative potential of -0.4 V, ramping up to 1.3 V and back down to -0.4 V in 8.5 ms. The waveform repeats at a frequency of 10 Hz. **B.** Jackson waveform begins at a positive potential of 0.2 V, sweeps up to 1.0 V and back down to -0.1V before returning to 0.2 V in 2.2 ms at 1000 Vs^{-1} and 10 Hz.

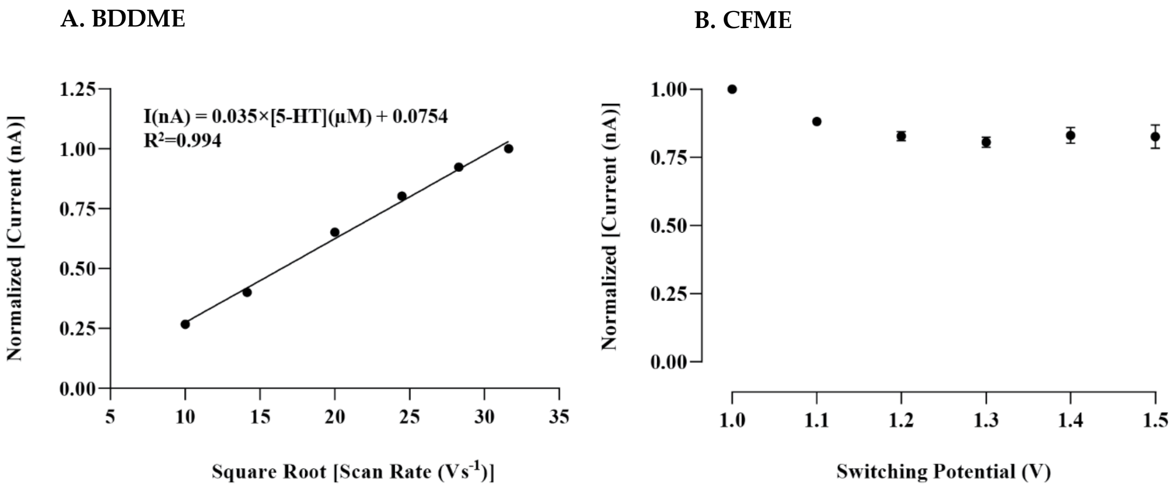

Figure S2. Further investigation of 5-HT oxidative response to scan rate at BDDMEs, and to increasing switching potential at inactivated CFMEs. **A.** 5-HT anodic peak response plotted against square root of scan rate to demonstrate possible diffusion-controlled kinetics at electrode surface, R^2=0.9939. **B.** Oxidative 5-HT responses at previously activated CFMEs with increasing switching potentials show a decreasing trend where 1.0 V has maximum response.

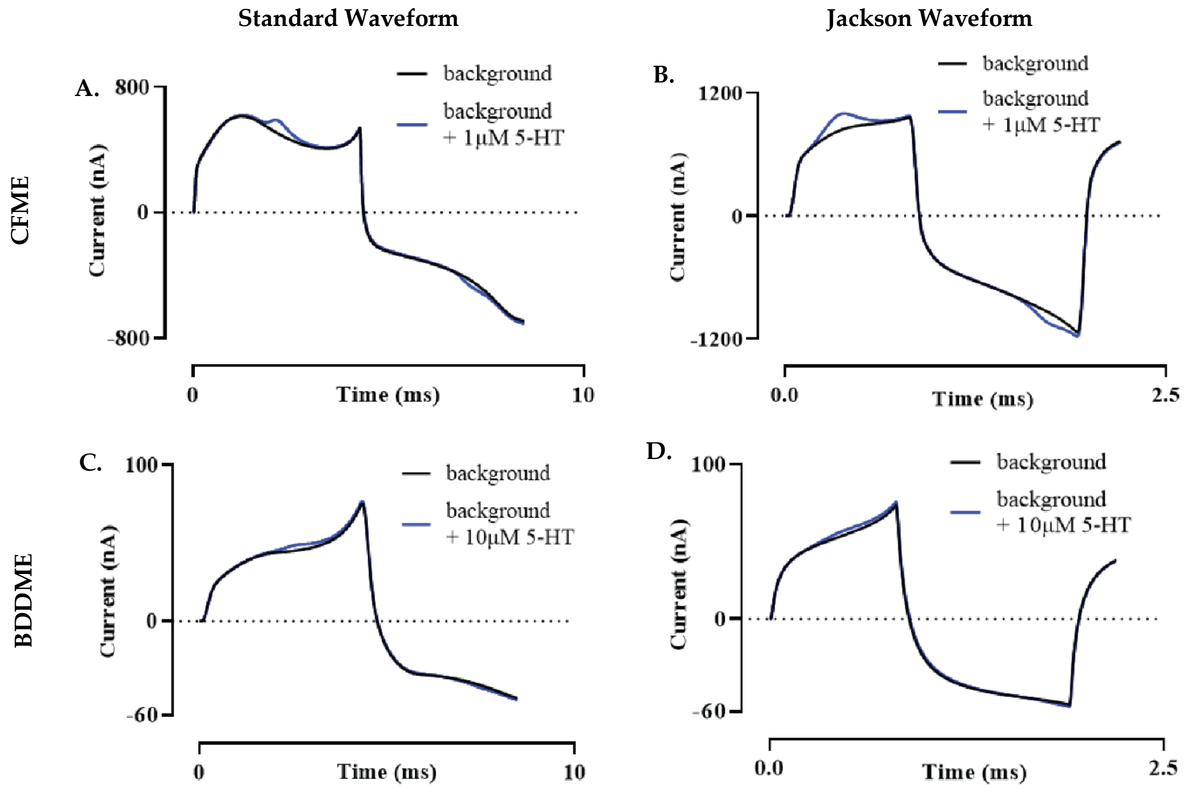

Figure S3. Background + faradaic currents at CFMEs and BDDMEs. **A. & B.** Faradaic current of 1 μM 5-HT current plotted over background current for CFME with standard and Jackson waveforms, respectively. **D. & C.** Faradaic current of 10 μM 5-HT plotted over background current for BDDME with standard and Jackson waveforms, respectively.

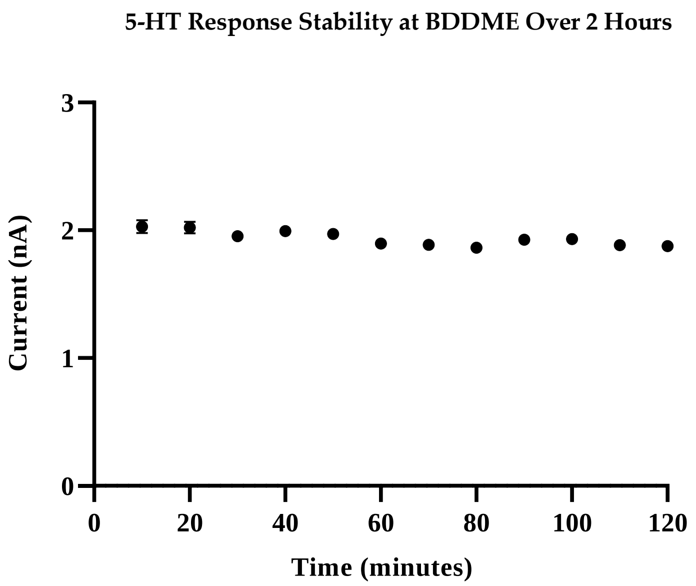

Figure S4. Response stability over 2 hours at the BDDME with the Jackson waveform. Current responses to injections of 10 μM 5-HT administered every 8-10 minutes for 120 mins (current variation < 10%). Data are represented as mean ± SEM for BDDME (*n*=1).
